# Developmental basis for flower sex determination and effects of cytokinin on sex determination in *Plukenetia volubilis* (Euphorbiaceae)

**DOI:** 10.1007/s00497-019-00382-9

**Published:** 2020-01-06

**Authors:** Yan Luo, Bang-Zhen Pan, Lu Li, Chen-Xuan Yang, Zeng-Fu Xu

**Affiliations:** 1 CAS Key Laboratory of Tropical Plant Resources and Sustainable Use, Xishuangbanna Tropical Botanical Garden, The Innovative Academy of Seed Design, Chinese Academy of Sciences, Menglun Mengla, 666303 Yunnan China; 2 Gardening and Horticulture Department, Xishuangbanna Tropical Botanical Garden, Chinese Academy of Sciences, Menglun, Mengla, 666303 Yunnan China; 3 Yunnan Academy of Biodiversity, Southwest Forestry University, Kunming, 650224 Yunnan China

**Keywords:** Monoecy, Sex determination, Euphorbiaceae, Unisexual flowers, Flower development, 6-Benzyladenine

## Abstract

*****Key message***:**

**Cytokinin might be an important factor to regulate floral sex at the very early stage of flower development in sacha inchi.**

**Abstract:**

Sacha inchi (*Plukenetia volubilis*, Euphorbiaceae) is characterized by having female and male flowers in a thyrse with particular differences. The mechanisms involved in the development of unisexual flowers are very poorly understood. In this study, the inflorescence and flower development of *P. volubilis* were investigated using light microscopy and scanning electron microscopy. We also investigated the effects of cytokinin on flower sex determination by exogenous application of 6-benzyladenine (BA) in *P. volubilis*. The floral development of *P. volubilis* was divided into eight stages, and the first morphological divergence between the male and female flowers was found to occur at stage 3. Both female and male flowers can be structurally distinguished by differences in the shape and size of the flower apex after sepal primordia initiation. There are no traces of gynoecia in male flowers or of androecia in female flowers. Exogenous application of BA effectively induced gynoecium primordia initiation and female flower development, especially at the early flower developmental stages. We propose that flower sex is determined earlier and probably occurs before flower initiation, either prior to or at inflorescence development due to the difference in the position of the female and male primordia in the inflorescence and in the time of the female and male primordia being initiated. The influence of cytokinin on female primordia during flower development in *P. volubilis* strongly suggests a feminization role for cytokinin in sex determination. These results indicate that cytokinin could modify the fate of the apical meristem of male flower and promote the formation of carpel primordia in *P. volubilis.*

## Introduction

The evolution of sex differentiation is a topic of general interest to biologists because it is one of the strategies for outbreeding and a vital source of genetic variation (Bawa 1980; Thomson and Brunet 1990). In flowering plant, sex determination is a developmental process that facilitates allogamy to support fitness and survival (van der Pijl 1978; Lloyd 1982). Sex determination systems in angiosperm have evolved independently numerous times suggesting that a great variety of developmental and genetic mechanisms for unisexual flower formation (Chuck 2010; Diggle et al. 2011). The mechanisms of sex determination have been investigated in some plants, such as maize, cucumber, kiwifruit, and persimmon, which are mainly affected by genetics, plant hormones, and environmental factors (Grant et al. 1994; Juarez and Banks 1998; Tanurdzic and Banks 2004; Adam et al. 2011; Bai and Xu 2013; Golenberg and West 2013; Li and Liu 2017; Henry et al. 2018; Akagi et al. 2018, 2019; Yang et al. 2019).

The pattern and process of unisexual flower development have been broadly categorized into two main types (Mitchell and Diggle 2005; Diggle et al. 2011). In type I, which is more frequently observed, flowers are bisexual at initiation and become unisexual by termination of the development of androecium or gynoecium. Mature flowers of this type have aborted reproductive organs. In type II, flowers appear to be unisexual from inception and only initiate androecium or gynoecium. Mature flowers of this type do not have aborted reproductive organs. Regarding the developmental mechanisms of sex determination in unisexual flowers, the reproductive organ abortion at a particular stage is critical in type I flowers, while the reproductive organ initiation is critical in type II flowers (Diggle et al. 2011). In plants with type I flower development, sex determination occurs at the particular developmental stage, when one of the reproductive organs is arrested. For example, the inappropriate pistil in male flowers of maize is arrested at an early stage by programmed cell death after initiation (Cheng et al. 1983; Dellaporta and Calderon-Urrea 1994; Calderon-Urrea and Dellaporta 1999). In plants with type II development, sex determination occurs at the particular developmental stage when reproductive meristem differentiates and reproductive organ initiates. In spinach (*Spinacia oleracea*) and *Thalictrum dioicum*, apical meristem of male flowers and female flowers become readily distinguishable after sepals initiation (Sherry et al. 1993; Di Stilio et al. 2010).

Plant hormones are essential factors for regulating sex determination in various plant species (Dellaporta and Calderon-Urrea 1993; Khryanin 2002; Yamasaki et al. 2005; Gerashchenkov and Rozhnova 2013). Feminization of male flowers by cytokinin treatment has been reported in several species, including species of *Vitis*, *Spinacia*, *Cannabis*, and *Pinus* (Negi and Olmo 1966; Chailakhyan and Khryanin 1978; Galoch 1978; Wakushima et al. 1996). Negi and Olmo (1966) reported that the exogenous application of cytokinin converted male flowers to hermaphroditic flowers in *Vitis vinifera*. An allelic difference in a gene in the cytokinin metabolic pathway is supposed to contribute to the sex determination of grape (Fechter et al. 2012). In kiwifruit, a cytokinin response regulator *Shy Girl* was found to be a potential sex determinant gene acting as the suppressor of carpel development (Akagi et al. 2018). In studying the role of hormones in relation to sex determination, it is obviously important to determine the effects of the exogenous hormones on flower development. However, the effects of exogenous hormones treatment at different developing stages in relation to sex determination are remarkably little known.

Euphorbiaceae is a particularly interesting family for the study of sexual differentiation as it is characterized by having unisexual flowers in monoecious or dioecious plants and displays great diversity in structure of flowers and inflorescences (Webster 1994). In this study, we focused on *Plukenetia volubilis*, commonly known as sacha inchi, which is native to the tropical South America (Gillespie 1993; Cardinal-McTeague et al. 2019) and is wildly cultivated as a potential oil plant in other tropical regions of the world (Wang et al. 2018). *P. volubilis* is a monoecious species with numerous male flowers, but single female flower in a thyrse. To improve sacha inchi seed yield, increasing the number of female flower is a practical and useful way. Application of exogenous cytokinins has been reported to convert male flowers to female flowers in *P. volubilis* successfully (Fu et al. 2014). In order to understand genetic mechanisms of sexual differentiation in *P. volubilis*, a number of recent studies are attempting to identify the sex determination related genes in *P. volubilis* (Fu et al. 2018; Hu et al. 2018). However, no study has compared the very early stages of male and female *P. volubilis* flower development to ascertain when and how the first morphological differences between the sexes appear. The effects of exogenous hormones treatment on the flowers at different developmental stages in relation to female flower initiation in this species remain to be elucidated.

The study of organogenesis of unisexual flower in Euphorbiaceae is extremely scarce, even for the most common and best-known species, such as castor (*Ricinus communis*) and rubber tree (*Hevea brasiliensis*). Here, we undertook a floral organogenesis study of *P. volubilis* to characterize the development of the two sexual flowers, document the differences between them, and to uncover the time of sex determination, then we applied exogenous 6-benzyladenine (BA), which is a synthesized plant growth regulator functioning as cytokinin, to modify flower sex in this species to reveal the effects of cytokinin on sex determination at the flower developmental level. The results revealed that sex determination occurs at early developmental stage, and exogenous cytokinin treatment can take effect on the meristem of male flowers to lead it to feminizing. This study shed light on the developmental mechanism of sex determination in *P. volubilis* and provided a better understanding of the factors that control primordium initiation within the male and female flowers.

## Materials and methods

Plants of *P. volubilis* were grown in a field at the Xishuangbanna Tropical Botanical Garden, Chinese Academy of Sciences, Yunnan Province, China (21° 54′ N, 101° 46′ E, 580 m in altitude). Developing inflorescences were collected in April 2018 and fixed in 50% ethanol. For light microscopy, tissue was dehydrated in an alcohol series and embedded in paraffin. Serial sections were taken 8 μm and stained with hematoxylin. The histological sections were observed and photographed under a NIKON ECLIPSE Ci microscope. For scanning electron microscopy observation, the samples were partially dissected in 80% ethanol under a Nikon stereo microscope (SMZ800N). The resultant material was dehydrated in an ethanol series and critical point dried with CO_2_ in a drier (LEIKA EM CPD 030). The samples were mounted on metallic stubs with carbon conductive adhesive tapes, sputter-coated with gold (Quorum Q150R S), and observed at 10 kV using a ZEISS EVO LS10 scanning electron microscope.

The size of flower bud and the apical meristem of flower primordia were measured from SEM photographs of both male (*N* = 15) and female (*N* = 15) flowers using Image J software. The diameter of the flower bud was established as the distance between the opposite sepal primordia. The length of the apical meristem of the androecium primordium or the gynoecium primordium was established as the distance between the two sinuses of the opposite sepal primordia, exclusive of sepal primordia. The area of the apical meristem of flower primordium was established as the area enclosed by the base circle of the androecium primordium or the gynoecium primordium exclusive of sepal primordia.

The BA treatment was carried out in April 2018, using 1-year-old *P. volubilis* trees. We selected 30 uniform branches that have been bearing inflorescence buds for each treatment. Stock solution (0.5 M) of BA (BA, Bio Basic Inc., Toronto, Ontario, Canada) was prepared, using 1 M NaOH solution as dissolvent. The BA working (180 μM, approximately 40 mg/l) solution with 0.05% (v/v) TWEEN 20 (Polysorbate-20, Shanghai Sangon Biological Engineering Technology & Services Co., Ltd., China) was sprayed onto branches with a hand sprayer, wetting the branches to the point of runoff (approximately 15 ml BA working solution per branches). Control branches were sprayed with 15 ml distilled water containing 0.05% (v/v) TWEEN 20 and NaOH, the concentration of which is equivalent to that in the working solution. Spraying was conducted once at dusk. The treated inflorescence buds were about 0.5 cm in length with female floral primordium and a few terminal male floral primordia (Fig. 7, 0 day). The inflorescence buds were labeled for sampling at 2-day intervals after treatment (DAT), for example 0, 3, 6 DAT, etc. Samples were fixed overnight in 50% ethanol and examined through the scanning electron microscope as above mentioned.

## Results

### Morphology of inflorescences and flowers

**Fig. 1:**
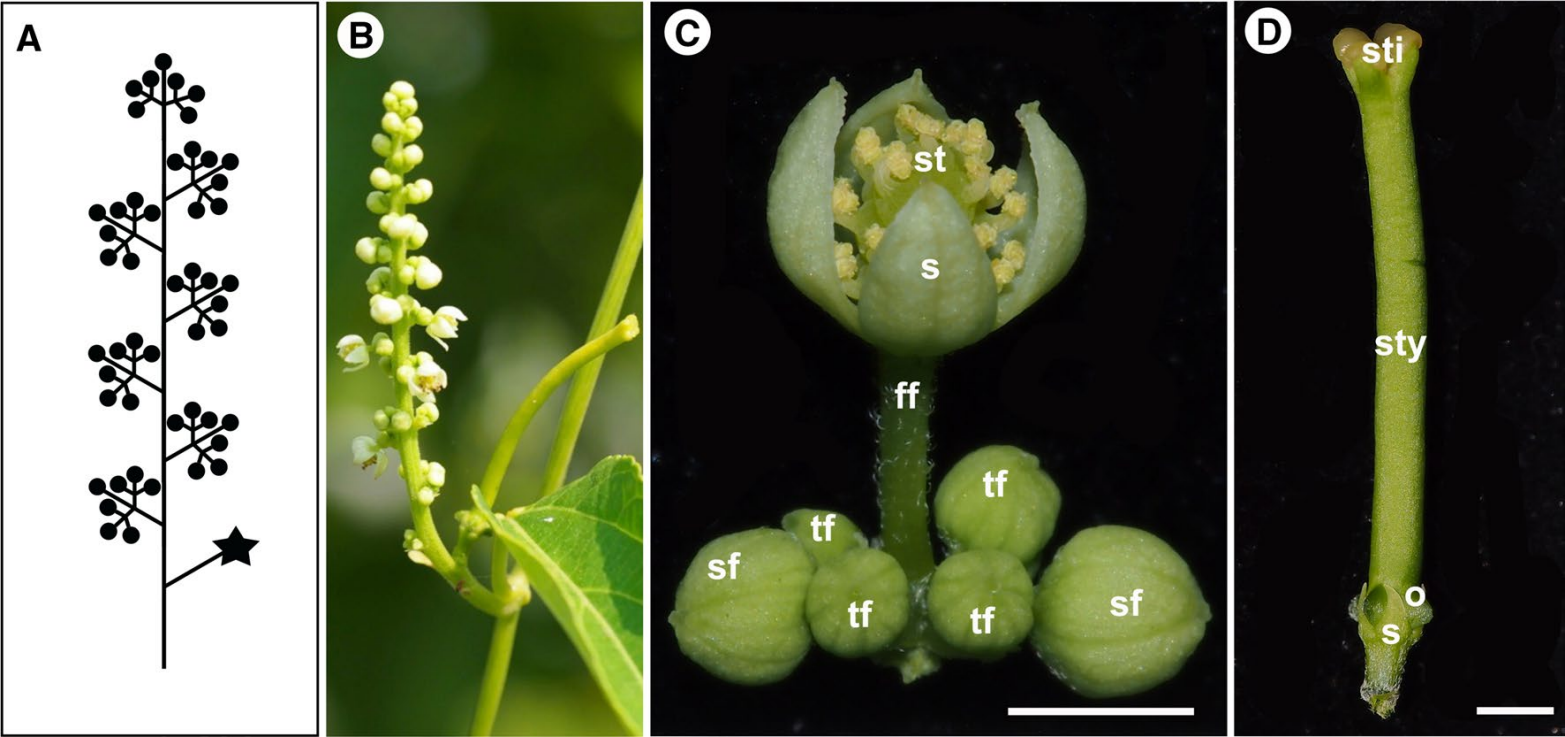
Morphology of reproductive organs in *Plukenetia volubilis*. **a** Schematic structure of inflorescence, male flowers (dots), a female flower (star). **b** Bisexual inflorescence at anthesis, a proximal solitary female flower, distal numerous male flowers clustered in cymules. **c** Male cymule with the first open flower. **d** Mature female flower. Abbreviations: s, sepals; st, stamen; o, ovary; sty, style; sti, stigma; ff, first flower; sf, second flower; tf, third flower; Scale bars: **c**, **d** = 2 mm

The inflorescence of *P. volubilis* is a bisexual thyrse often with coflorescences occurring in a lateral axis (Fig. 1a, b). The sex distribution within the inflorescence of *P. volubilis* is not random. It consists of a main axis bearing a series of spirally arranged primary bracts that subtend one or two female flowers and numerous lateral male cymules with 3–7 flowers per cymules (Fig. 1a, b). The number of flowers in one inflorescence may vary from 20 to hundreds.

The flowers of *P. volubilis* are apparently monochlamydeous without petals (Fig. 1c, d). The differences between the sexes are obvious in the mature flowers. Female flowers and male flowers are quite different in appearance. The male flowers are small, about 3 mm in diameter and 5 mm in length. The four or five greenish-yellow sepals are valvate, obovate, and fused into a bell-shaped calyx before anthesis. There are 16–30 subglobose stamens with stout conical filaments (Fig. 1c). Pistillodes and glandular disk segments are absent. The female flowers are quite unusual with a distinct gynoecium, about 3 mm in diameter and 30 mm in length, pointing toward the apex of the inflorescence. The four green sepals are quite small, alternate, triangular, and separated. The gynoecium is composed of 4–7 fused carpels. The 4–7 styles are mostly fused into a long and massive cylindrical stylar column. The green stylar column is 15–30 mm long. The ovary is 4–7 locular and superior, 4–7 angled to deeply 4–7 lobed, each angle or lobe with a laterally compressed wing. Each locule has only one ovule. Staminoides and glandular disk segments are absent (Fig. 1d).

### Development of the inflorescences

**Fig. 2:**
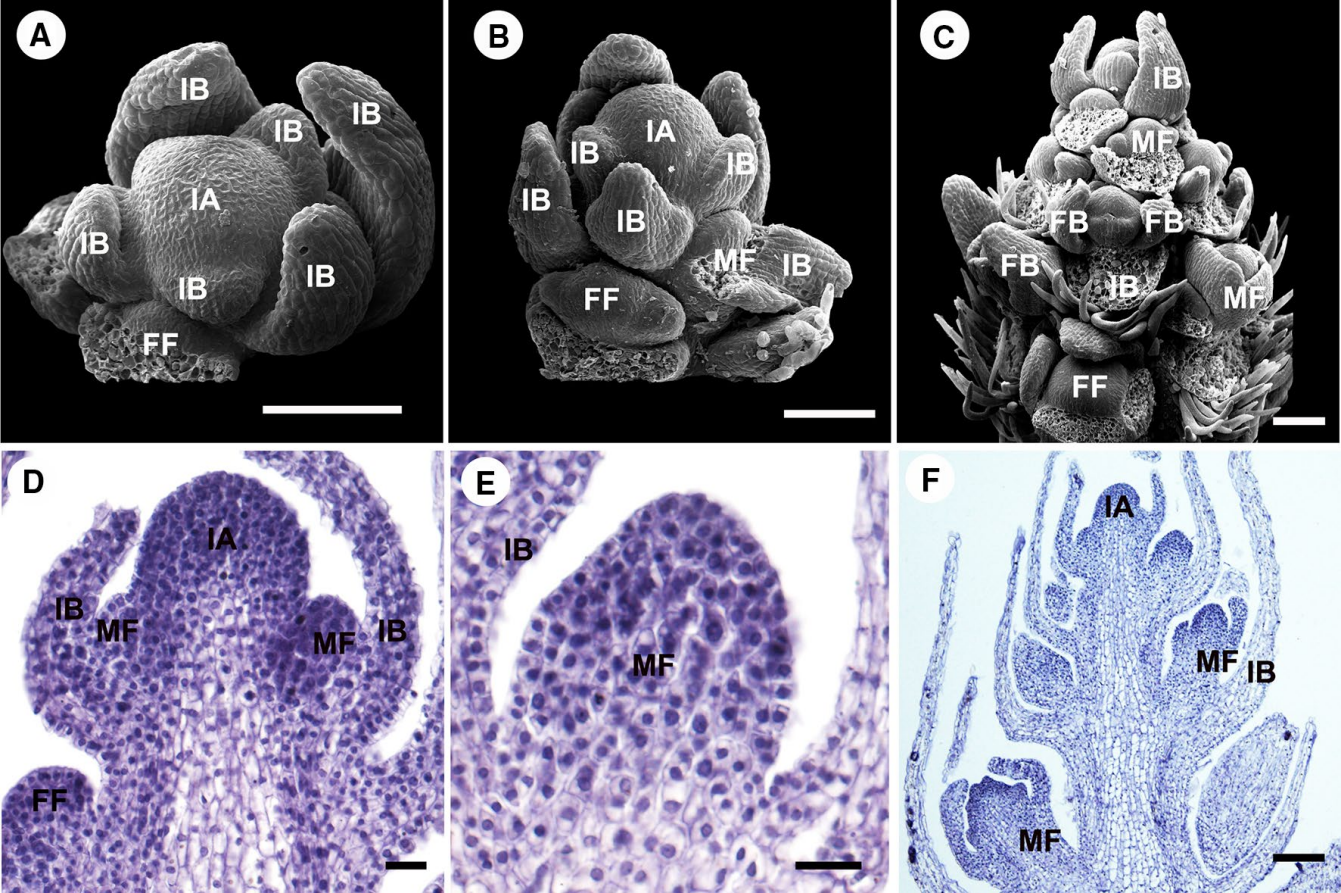
The development of inflorescence in *Plukenetia volubilis*. Scanning electron micrographs of the development of inflorescence in *P. volubilis* (**a**–**c**), light microscopy of the development of inflorescence in *P. volubilis* (**d**–**f**). **a** Frontal view of an inflorescence with a female flower primordium. **b** Lateral view of an inflorescence with female and male flower primordia. **c** Lateral view of an inflorescence with floral buds in different developmental stages, spirally arranged along the main axis, containing one female flower and groups of terminal male flowers of male cymules; some of lateral bracts and flower bracteoles were removed. **d** Longitudinal section of an inflorescence meristem at the same stage as shown in (**a**), where the female and male flower primordia can be observed. **e** Longitudinal section of an early male flower primordium. **f** Longitudinal section of an inflorescence meristem at the same stage as shown in (**c**), where the female and male flower primordia can be observed. Abbreviations: IA, inflorescence apex; IB, inflorescence bract; FB, floral bract; FF, female flower primordium; MF, male flower primordium. Scale bars: (**a**–**c**, **f**) = 100 µm; (**d**, **e**) = 20 µm

*P. volubilis* initially produces an indeterminate thyrse consisting of abaxial bracts with cymose inflorescences in their axils. The abaxial bract primordia of the inflorescence are initiated spirally on the flanks of the inflorescence apex (Fig. 2a). Development along the main inflorescence axis proceeds acropetally. Soon after initiation of the bract primordia become crescent shaped, the concave side of the crescent opening upward (Fig. 2b). The flower primordia are initiated in the axils of the abaxial bracts. The female flower primordium is the first to initiate in the axil of the first bract of the inflorescence (Fig. 2b, d). The cymules containing male flowers are initiated in the axil of the later bracts along the inflorescence axis afterward (Fig. 2c, e, f). The terminal male flower is the first to initiate differentiation in each male cymule. As the inflorescence ages, the inflorescence apex decreases in size relative to the surrounding bracts and finally produces a terminal male flower and ceases growth.

### Female flower development

Based on the differentiation steps involved in the formation of all *P. volubilis* floral organs by SEM and histological section investigation, we divided the process of flower development into eight stages roughly following the scheme established for the model plant *Arabidopsis thaliana* summarized by Smyth et al. (1990). The characteristics of both male and female flower development stages are given in Table 1.

**Table 1:** Summary of stages of flower development in *Plukenetia volubilis*, listing the morphological events that define the beginning of each stage and the mean size of the floral meristem or the flower bud

Stage	Female flower		Male flower	
	Morphological events	Diameter of flower bud (mm)	Morphological events	Diameter of flower bud (mm)
1	Initiation of floral meristem	0.05–0.06	Initiation of floral meristem	0.04–0.06
2	Emergence two bracteoles	0.09–0.11	Emergence of two bracteoles	0.06–0.08
3	Emergence of sepal primordia	0.21–0.26	Emergence of sepal primordia	0.12–0.17
4	Initiation of carpel primordia	0.25–0.33	Initiation of stamen primordia	0.18–0.25
5	Enlargement of carpel primordia and enclosure to form pistil primordium	0.40–0.48	Enlargement of stamen primordia as balls	0.36–0.45
6	Initiation of ovular meristem	0.65–0.72	Stamen stalked at the base, and differentiated to produce cone-shaped anther	0.52–0.57
7	Initiation of nucellus and integument in ovule; pistil primordium elongates and differentiates into style	0.80–0.92	Locules differentiated in stamen and mitosis in anthers	0.60–0.65
8	Stigmas are formed at the end of pistil; ovules expand	1.00–1.30	Stamen increase in size, meiosis in anthers, pollen appears in pollen sac	0.64–0.72

**Fig. 3:**
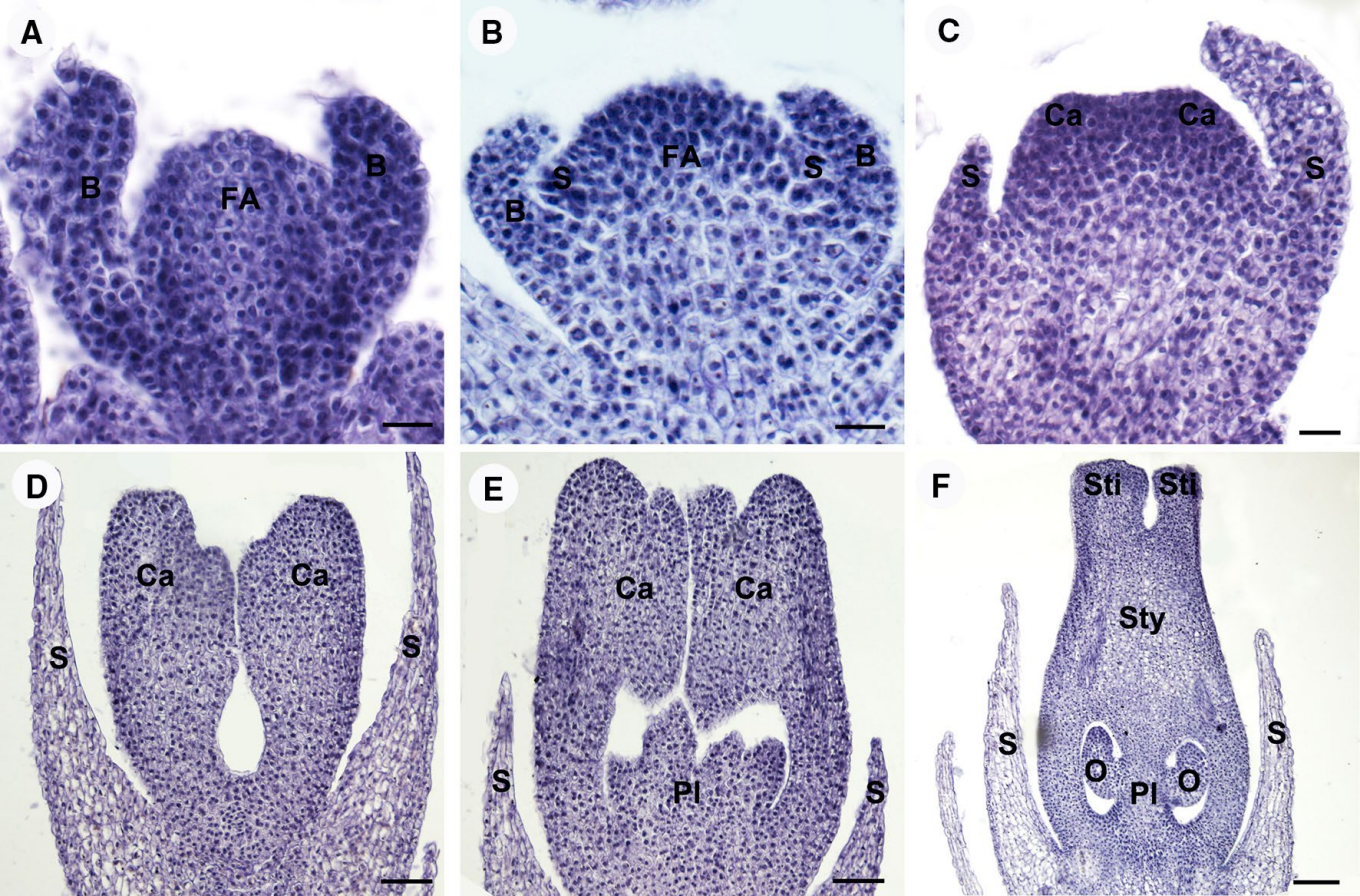
Longitudinal sections of female flower meristem showing the development of female flower in *Plukenetia volubilis* under a light microscopy. **a** The floral meristem initiation at stage 2. **b** The sepal primordia initiation at stage 3. **c** The cube-shaped carpel primordia initiation at stage 4. **d** The carpel primordia united with its enclosed central cavity forms the pistil primordium at stage 5. **e** The locules formation at stage 6. **f** A complete pistil development at stage 8. Abbreviations: FA, floral apex; B, bracteole; S, sepal primordium; Ca, carpel primordium; Pl, Placenta; O, Ovule; Sty, Style; Sti, Stigma. Scale bars: (**a**–**c**) = 20 µm; (**d**, **e**) = 50 µm; **f** = 100 µm

**Fig. 4:**
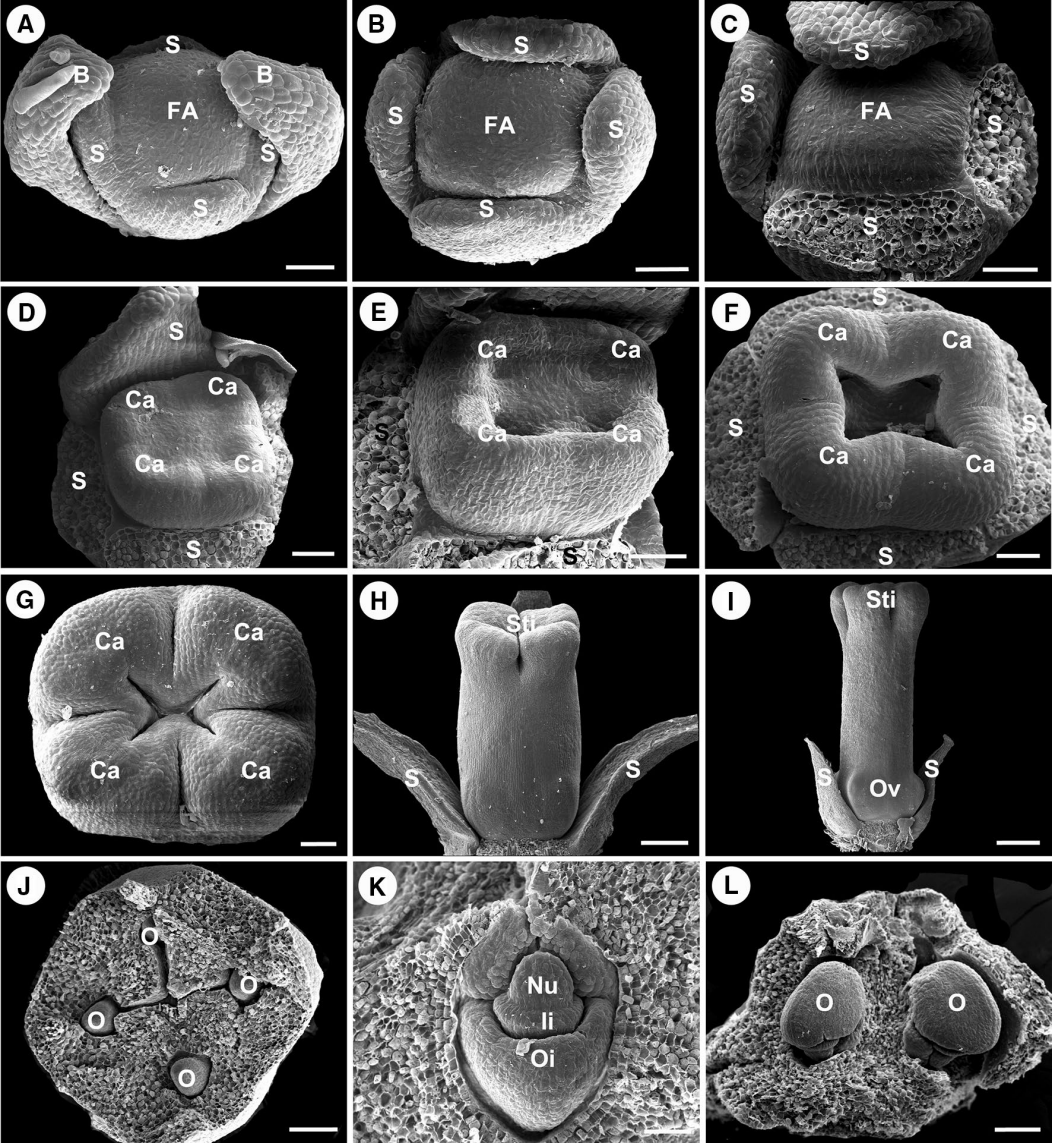
Scanning electron micrographs of the development of female flower in *Plukenetia volubilis*. **a**–**c** Female meristem in front view at the third stage of the sepal formation; **a** The four sepal primordia are initiated in a whorl pattern; **b** The developing sepal primordia with similar size and shape; **c** The flatten central floral meristem. **d**–**f** Frontal view of a female meristem showing the formation of the carpel primordium at stage 4; **d** A cube-shaped carpel primordium initiation; **e** Four marginal depressions become apparent; **f** A star-shaped ring is evident. **g** Floral meristem in front view at stage 5 showing the fusion of the carpel primordia for the formation of the gynoecium primordium. **h** Floral meristem in lateral view at stage 7 showing the stigma formation. **i** Floral meristem in lateral view at the final stages of the stigma and ovary formation. **j**–**l** Frontal view of transection of ovary primordium showing the ovules formation. **j** Four ovule primordia can be observed; **k** Lateral view of developing ovule with outer integument, inner integument and nucellus; **l** The developed ovule. Abbreviations: FA, floral apex; B, bracteole; S, sepal primordium; Ca, carpel primordium; Sti, Stigma; Ov, ovary; O, ovule primordium; Oi, outer integument; Ii, inter integument; Nu, nucellus. Scale bars: (**a**–**g**, **k**) = 50 µm; **h** = 200 µm; (**i**) = 500 µm; (**j**, **l**) = 100 µm

The first stage of floral development becomes visible from the axil of the basal bract of the inflorescence. The floral meristem is initiated as an ellipsoidal bulge of approximately 55 μm in diameter (Fig. 2a, d). At stage 2, the floral primordium is widened to produce a transversely elongated primordium. As growth continues the primordium becomes more pointed on two sides. These two points are the bracteoles at the time of their initiation. The alternate paired bracteoles are the first organs to be produced (Fig. 3a). The elongation of the bracteoles is followed by the formation of trichomes on their abaxial surface, covering the flower meristem. The central portion of the main dome would give rise to the subsequent floral organs. In stage 3, the central floral meristem enlarges, begins to flatten apically, and assumes a rounded, square appearance in polar view. As growth continues, the floral primordium becomes flatten and the flanks of the square become more pronounced. The rounded flanks of the floral primordium are the sites of sepal initiation (Fig. 3b). The four sepal primordia are initiated in a whorl pattern (Fig. 4a). The size of the apical meristem of flower primordia (the gynoecium primordium) is 139 ± 22 μm in length and approximately 0.015 mm^2^ in area measured as Fig. 4a (FA, floral apex). The order of appearance of four sepal primordia seems to be at the same rate (Fig. 4a–c). The sepal primordia enlarge somewhat before gynoecial initiation. The developing sepals with similar size and shape are separate but not confluent before anthesis. At stage 4, the whole flower primordium interior to the sepals continues to enlarge to produce a cube-shaped tissue during sepal initiation (Figs. 3c, 4d). Then the periphery of the flower meristem grows upward, and four (sometimes up to seven) marginal depressions become apparent, and a star-shaped ring of tissue that surrounds a central depression is evident (Fig. 4e, f). At stage 5, this ring primordium is composed of four (sometimes up to seven) partially distinct carpel primordia united below into a cylinder of tissue surrounding a central cavity (Figs. 3d, 4g). The cylinder with its enclosed central cavity forms the pistil primordium. The central cavity is evident inside the young ovary in longitudinal section (Fig. 3d). At stage 6, continued growth of the gynoecial primordia produces four locules by simultaneous differentiation of the ovary wall at the closure of the carpels. The body of the gynoecial primordia produces the septa and the central axis of the ovary through their fusion at the center of the ovary (Fig. 3e). The locules arise from an expansion of the spaces between gynoecial primordia. In each locule, the ovule primordium bulged outward, forming an oval shape (Fig. 4j). At stage 7, the style and stigma form from the apical portions of the gynoecial primordia. The four carpel primordia grow upward and fuse along their lateral margins to produce a massive cylindrical style capped by a four-lobed stigma primordium (Fig. 4h). A complete pistil is observed at stage 8, with four stigmas; the styles are quite elongated (Figs. 3f, 4i). With elongation and complete development of the gynoecium, the styles become long and massive. The ovule primordium produces outer integument, inner integument, and nucellus in turn (Fig. 4k). As the ovules enlarge, four protrudes are formed like wings outward of ovary (Fig. 4i). The four ovules are in the shape of a compressed sphere (Fig. 4l).

### Male flower development

Lateral male cymule primordia are initiated on the flanks of the inflorescence apex in the axil of each abaxial inflorescence bract. Following initiation of the paired bracteoles and the young terminal flower, a secondary pair of axillary floral bud is initiated rather low at the base of the first flower as a small lobe. Tertiary pairs of flowers are initiated at the base of each of the secondary flowers as well as at the base of the first flower. This pattern is reiterated until there are 7–15 male cymules in an axillary inflorescence. Since pedicels are very short, the male cymule is a tight cluster. Typically, a small bract forms in each male cymule. The cymules containing male flowers show later development compared with the ones with female flowers.

**Fig. 5:**
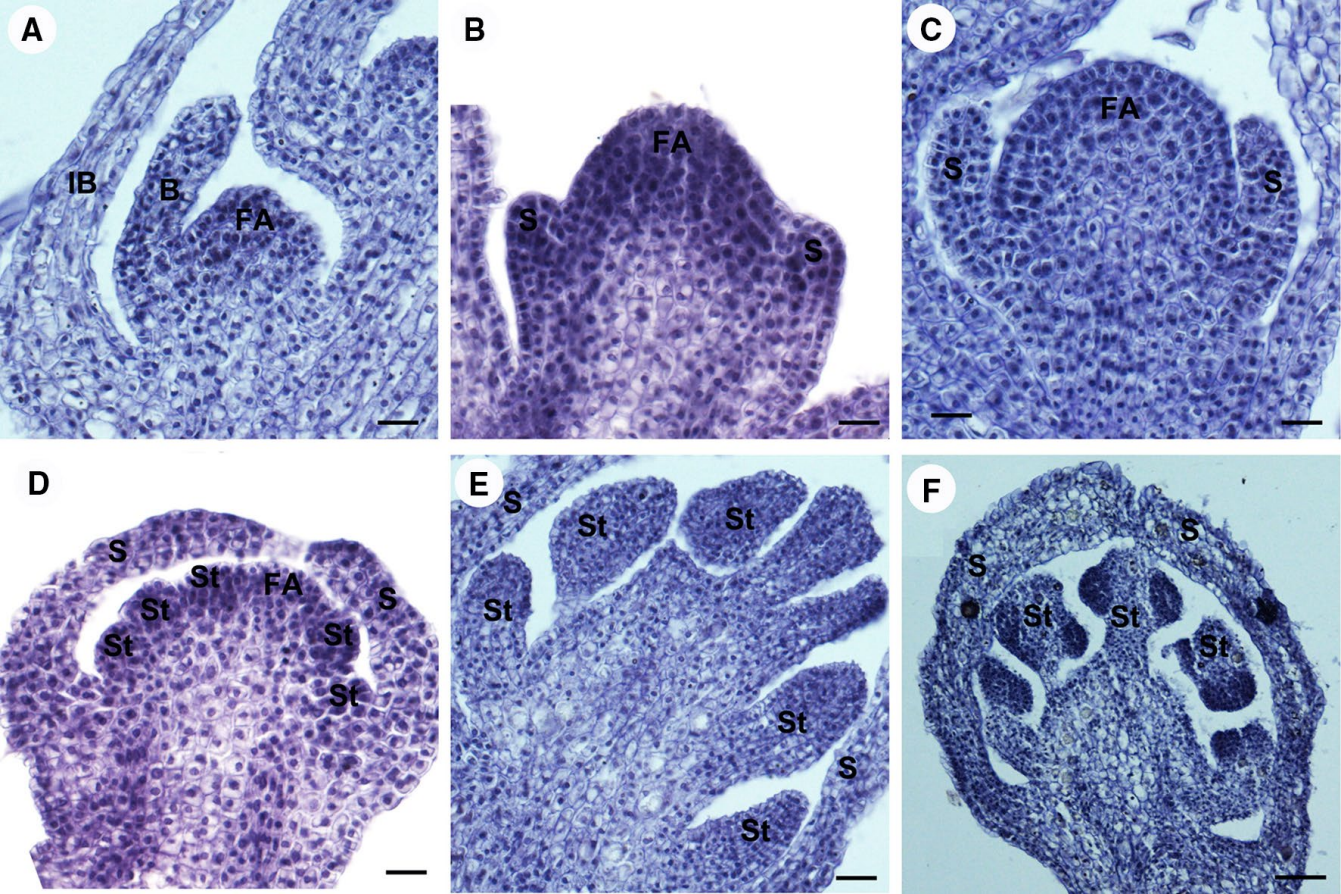
Longitudinal sections of male flower meristem showing the development of male flower in *Plukenetia volubilis* under a light microscopy. **a** The floral meristem initiation at stage 2. **b** The sepal primordia initiate at stage 3. **c** The sepal primordia elongate. **d** The stamen primordia are initiated at stage 4. **e** The stalked and bulge-shaped stamen formation at stage 5. **f** The anther development at stage 6. Abbreviations: FA, floral apex; IB, inflorescence bract; B, bracteole; S, sepal primordium; St, stamen primordium. Scale bars: (**a**–**e**) = 20 µm; (**f**) = 50 µm

**Fig. 6:**
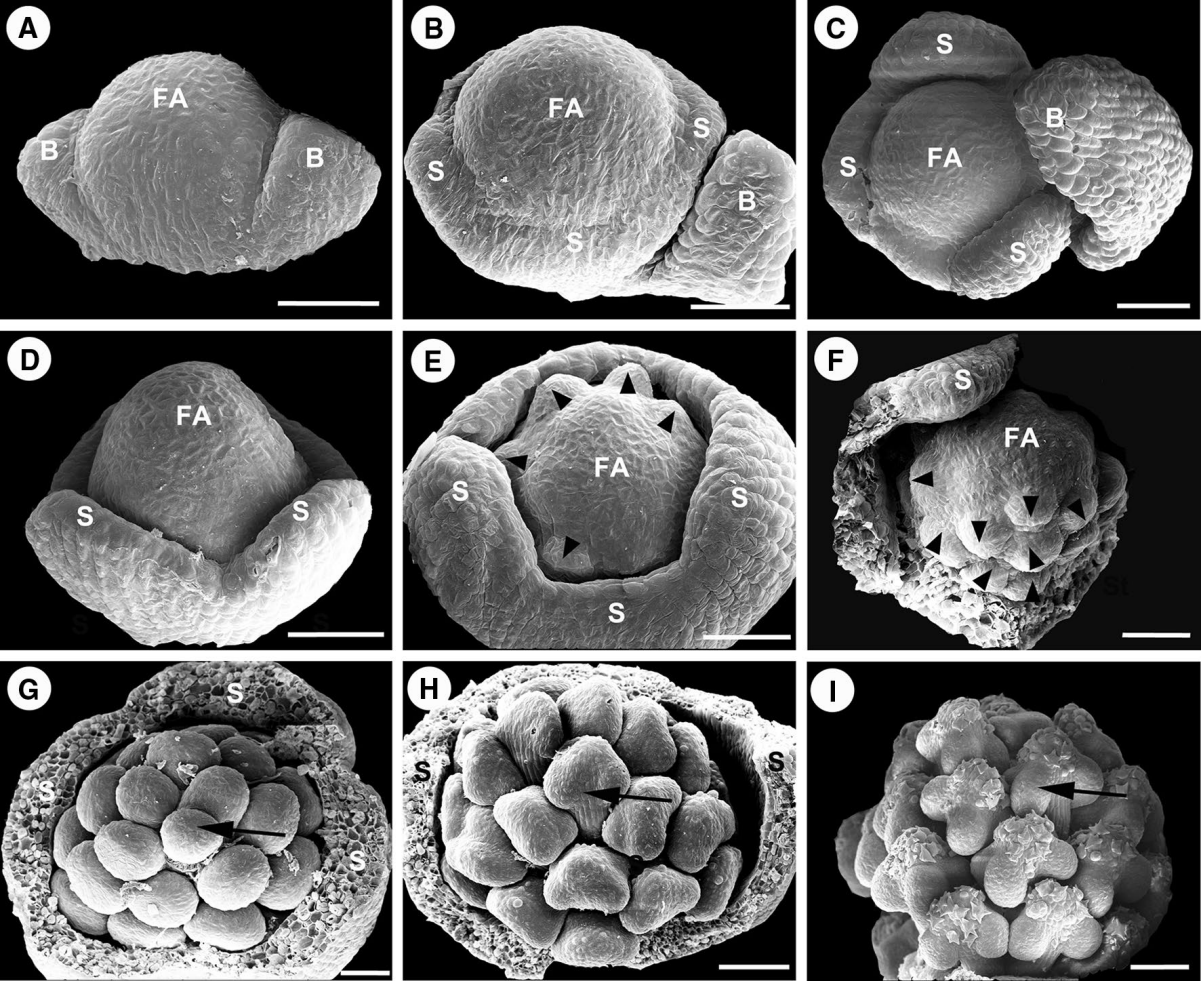
Scanning electron micrographs of the development of male flower in *Plukenetia volubilis*. **a** The male meristem in lateral view at stage 2, where two alternate bracteoles primordia are initiated. **b** The male meristem in front view at stage 3, where four sepal primordia are initiated in a whorl pattern. **c** The developing sepal primordia with similar size and shape. **d** The developing sepal primordia elongate to produce the confluent calyx. **e** Frontal view of a male meristem showing the formation of the stamen primordium at stage 4, black arrowheads indicating stamen primordia. **f** Lateral view of a male meristem showing the formation of the stamen primordium at stage 4, black arrowheads indicating stamen primordia. **g** Floral meristem in front view at stage 5 showing the developing stamen primordia with stalk, a black arrow indicating stamen primordium. **h** Floral meristem in front view at stage 6 showing the anther developing, a black arrow indicating stamen primordium. **i** Floral meristem in front view at the final stages of a complete stamen formation, a black arrow indicating stamen primordium. Abbreviations: FA, floral apex; B, bracteole; S, sepal primordium. Scale bars: (**a**–**g**) = 50 µm; (**h**–**i**) = 100 µm

The characteristics defining eight stage of male flower development are given in Table 1. The first two stages of male flower development are same as female flower. The flower meristem arises from the axil of abaxillary bract of the inflorescence. After two alternate bracteoles primordia initiate, the central floral meristem produces other floral organs (Figs. 5a, 6b). At stage 3, it can be observed in *P. volubilis* that four sepal primordia are initiated in what appears to be a whorl pattern at the base of the floral apex (Figs. 5b, 6b). The size of the apical meristem of flower primordia (the androecium primordium) is 110 ± 16 μm in length and approximately 0.009 mm^2^ in area measured as shown in Fig. 6b (FA, floral apex). The confluent sepal primordia enlarge and elongate as a whole to produce the majority of the confluent calyx below the tips of sepals before androecial initiation (Figs. 5c, 6c, d). During the elongation of the calyx, four sepal primordia are continuously combined as a whole and not to be free, resulting in a fused calyx with free tips of sepals covering the floral apex. At stage 4, flower meristem internal to the sepal primordia begins to differentiate. The stamen primordia as bulges may be observed in a centripetal formation. The earliest stamen primordia are initiated in the margin (Fig. 6e). The later stamen primordia initiated at the apex of the apical meristem and are quickly covered by the growth of the sepals (Fig. 6f). At stage 5, all of stamen development is at the same rate. Followed by cell divisions, the primordia of the stamens are ball-shaped. The stamen primordia are active with dark staining in the nuclei (Fig. 5d). At stage 6, the stamen primordia show elongation and become stalked at the base. The primordia of the stamens are changed from ball-shaped to cone-shaped. The stalk and the cone would eventually development into the filament and anther (Figs. 5e, 6g), respectively. At stage 7, four protrusions are formed at the bottom of anthers, forming the thecae of the stamen. The cell division in adaxial side of the anther is densely stained and a bilateral structure is established with locules (Figs. 5f, 6h). At the post-anthetic stage 8, the stamens show short and stout filaments, and the anthers become completely formed, with pollen grains that are initiating their own development. The anthers are characterized by flattening of the thecae, with some irregular fragments that expand over the anthers (Fig. 6i). We do not know what substances are. Soon afterward, anthesis occurred.

### Induction of carpel primordium in male flowers by BA

**Fig. 7:**
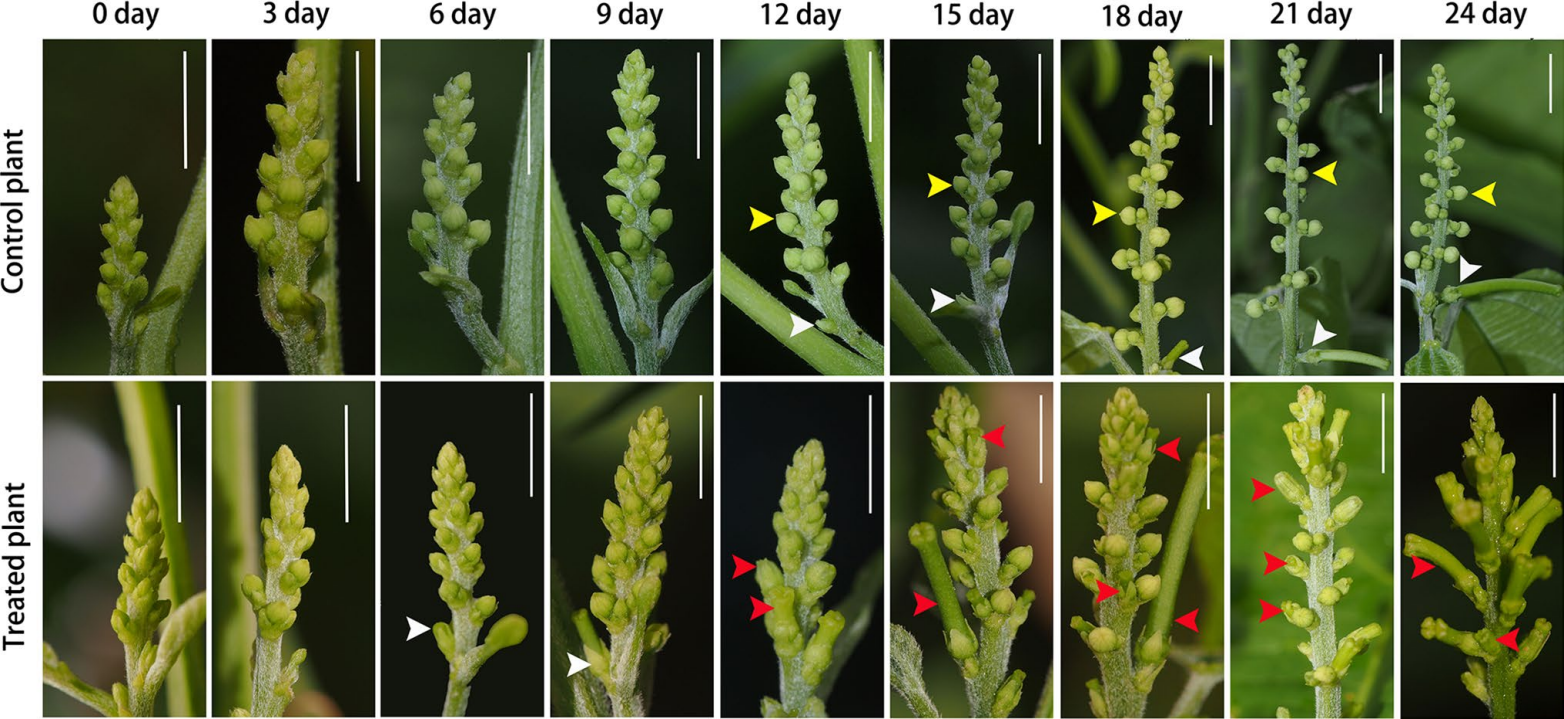
BA-treated inflorescence in *Plukenetia volubilis*. White arrow indicating female flower buds, yellow arrows indicating male flower buds, red arrows indicating induced female flower buds induced by BA. Scale bars: 5 mm

**Fig. 8:**
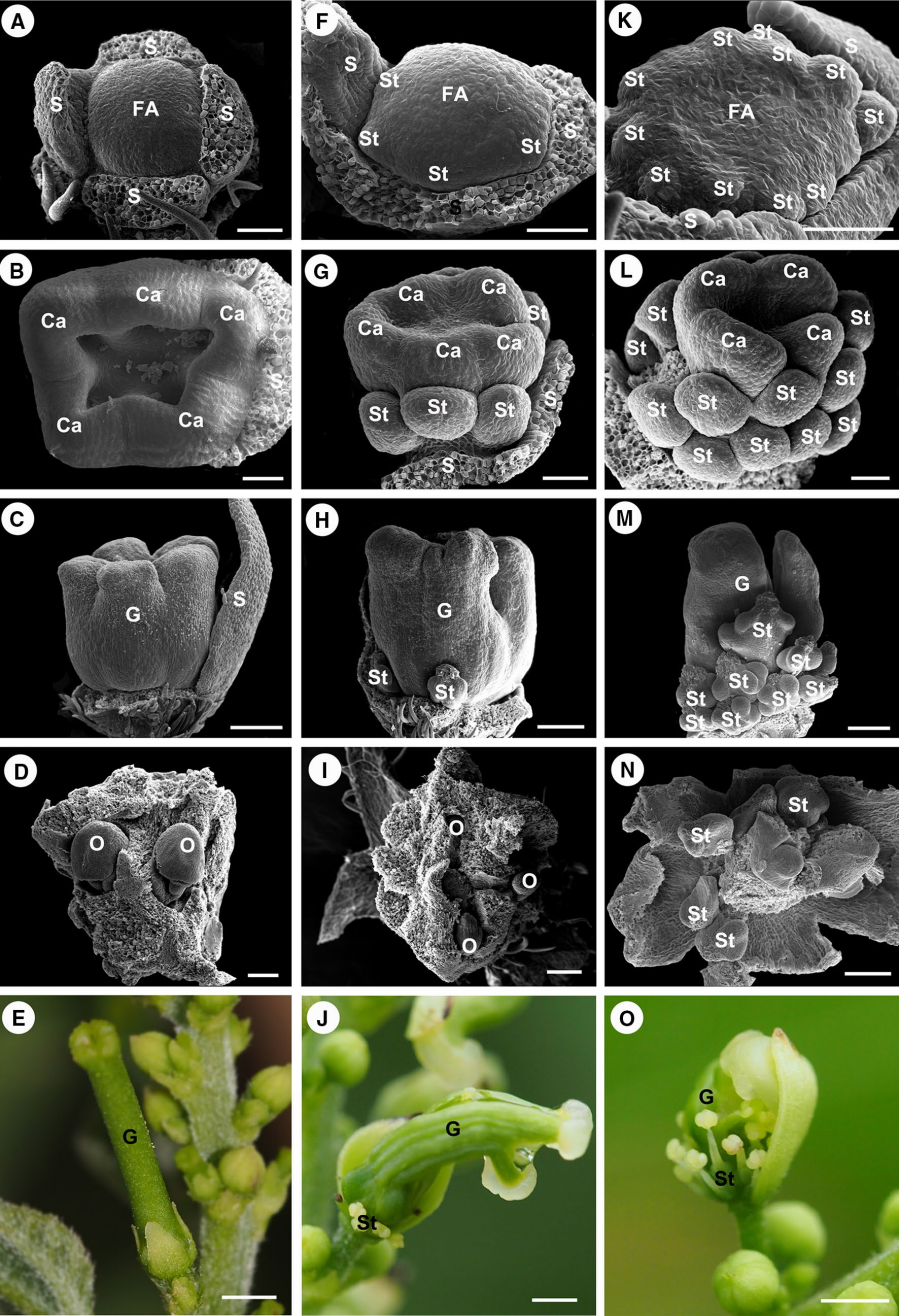
Three patterns of flower development in BA-treated male flower meristem in *Plukenetia volubilis*. **a**–**e** Normal female flower development in treated flower meristem; **a** a flat floral apex is formed; **b** the carpel primordia are initiated; **c** the gynoecium primordium is formed; **d** the developed ovules in the ovary; **e** a normal female flower is formed; **f**–**j** hermaphroditic flower development in treated flower meristem; **f** a few stamen primordia are initiated at the base of floral apex; **g** the carpel primordia are initiated at the central of floral meristem; **h** the gynoecium primordium is formed with stamens at the base; **i** the developed ovules in the ovary; **j** a hermaphroditic flower is formed with fertile stamens and pistil. **k**–**o** Abnormal hermaphroditic flower development in treated flower meristem; **k** numerous stamen primordia are initiated; **l** the carpel primordia are initiated at the central of floral meristem; **m** an abnormal gynoecium is formed with numerous stamens at the base; **n** no ovules found in the ovary; **o** an abnormal hermaphroditic flower is formed with fertile stamens. Abbreviations: FA, floral apex; S, sepal primordium; Ca, Carpel primordium; St, stamen primordia; G, gynoecium primordium. Scale bar: (**a**, **b**, **f**, **g**, **k**, **l**) = 50 µm; (**d**, **i**, **n**) = 100 µm; (**c**, **h**, **m**) = 200 µm; (**e**) = 2 mm; (**j**, **o**) = 5 mm

In the BA-treated inflorescence 3, 6, and 9 days after treatment, no female flowers appeared at the position where the male flowers would normally be located (Fig. 7). The developmental pattern of male flowers was just like normal male flowers, as we show in Fig. 6, and no carpel primordium initiation in male flowers observed. In the BA-treated inflorescence 12 days after treatment, carpel primordium appeared at the secondary and tertiary male flowers. In the BA-treated inflorescence, 15, 18, 21, and 24 days after treatment, more and more carpel primordia could be observed at the first, secondary, and tertiary male flowers (Fig. 7). Three different patterns of carpel development were found in BA-treated male cymules. The first pattern is normal carpel development as observed in female flowers (Fig. 8a–e). In this pattern, a flat floral apex initiated (Fig. 8a), and then normal carpel fused as pistil primordium without any stamen primordia appearing (Fig. 8b) and then developed into normal pistil (Fig. 8c, d). These mature flowers seemed normal female flowers (Fig. 8e). The second pattern is carpel initiation from early development stage of stamen primordia (Fig. 8f–j). In this pattern, a few stamen primordia had been initiated at the base of floral apex, while the central of floral apex became flat and carpel primordia initiated (Fig. 8f, g), then stamen primordia developed into stamens at the base of flower and not well-developed pistil formed at the central part of flower (Fig. 8h, i). These mature flowers seemed hermaphroditic flowers with fertile stamens and pistils (Fig. 8j). The third pattern is carpel initiation from later stamen primordia development stage (Fig. 8k–o). In this pattern, numerous stamen primordia had been initiated, while carpel primordia initiated from the limited space at the central of floral apex (Fig. 8k, l), and then more normal stamens and an abnormal pistil formed subsequently (Fig. 8m). These flowers were morphologically deformable hermaphroditic flowers with incurved style and without ovules (Fig. 8n, o).

## Discussion

### Developmental mechanisms of sex differentiation in *P. volubilis*

There are many different developmental mechanisms underlying unisexual flower development. Diggle et al. (2011) recognized four stages of sexual differentiation as “stage 0,” before the initiation of stamen or carpel primordia; “stage 1,” early in stamen or carpel development; “stage 2,” pre-meiosis; and “stage 3,” post-meiosis. In type I species, flowers are bisexual at initiation and become unisexual by termination of the development of functional sex organ during various developmental processes from stage 1 to stage 3. As observed in *Vernicia fordii*, *Jatropha curcas*, the female flowers presented bisexual characteristics with staminodes existing. The stamen primordia are initiated in the female flower, but arrested after the pistil primordia development (Liu et al. 2008; Wu et al. 2011; Zhang et al. 2012; Pan et al. 2016; Mao et al. 2017). In a number of species, with type II unisexual flowers, including *Cannabis sativa* (Mohan Ram and Nath 1964), *Mercurialis annua* (Durand and Durand (1991), *S. oleracea* (Sherry et al. 1993), and *Humulus lupulus* (Shephard et al. 2000), only one sex organ is formed during ontogeny and sex differentiation occurs before initiation of stamens and carpels (at “stage 0”).

**Fig. 9:**
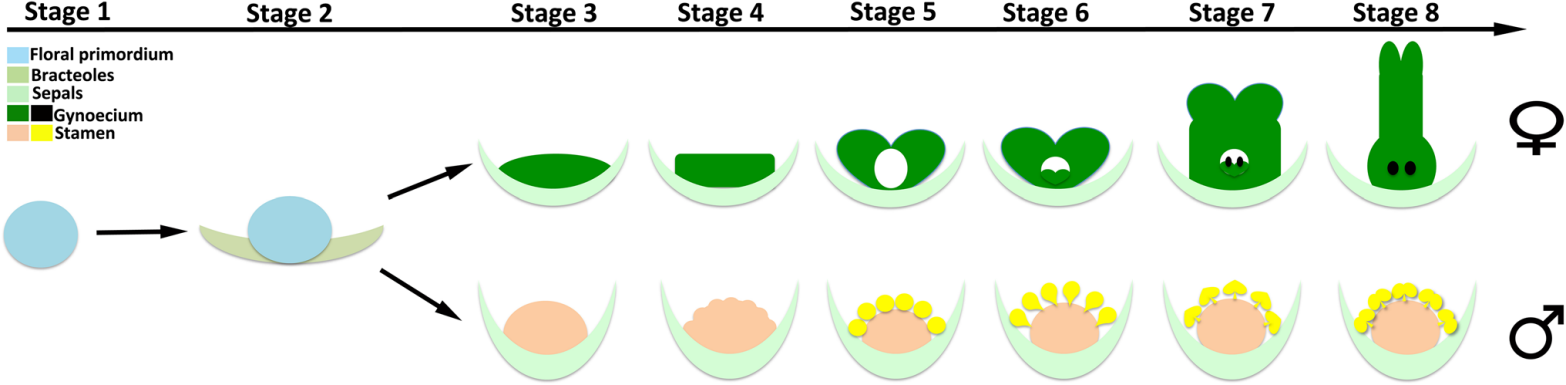
Schematic illustration for developmental stages of *Plukenetia volubilis* showing major events occurring and developmental difference in male and female flowers. Different colors represent floral organs, and the bracteoles were not shown from stage 3 to stage 8

According to our study, both female and male flowers of *P. volubilis* belong to type II unisexual flowers. We divided eight developmental stages for both male and female flower development and compared developmental differences between male and female flowers as we showed in Fig. 9 and Table 1. At the earliest two stages, the female and male primordia developmental sequences are not significantly different. The size and shape of the two kinds of primordia are indistinguishable (Fig. 2a, b). The cell size and cell shape are the same in both types of primordia (Figs. 3a, 5a). The flower primordia of *P. volubilis* are histologically indistinguishable at inception, suggesting a common primordia type for the flower with different sex. The two flower types noticeably diverge in their development after sepal primordia initiation, and their shapes and size differ significantly thereafter. SEM showed that the female and male flowers could be distinguished since the sepal producing onward (Figs. 4, 6). After the sepal primordia elongation, the male and female flower meristems began to exhibit some developmental differences, particularly morphological changes in the central dome of the flower primordium. For male flowers, the central floral meristem was hemispherical (see Fig. 6d, ca. 110 μm in length), from which small bulges, i.e., stamen primordia appeared (Fig. 6e), while for female flowers, the central floral meristem became flat (see Fig. 3c, ca. 139 μm in length), from which the carpel primordia initiated. We assumed this stage is to be responsible for sex differentiation in *P. volubilis*, as sexual differentiation of “stage 0” recognized by Diggle et al. (2011).

Obviously, the production of both sexes presenting in *P. volubilis* plants is also depended on floral meristem position in the inflorescence. Typically, after the initiation of inflorescence with primary bracts, the floral primordium appears at the axil of the primary bract on the inflorescence rachis. The most basal floral primordium is female, and all of the other distal floral primordia are male. The difference in the position of the floral primordium in the inflorescence and in the time of the floral primordium being initiated, however, implies that flower sex is determined earlier. This determination event probably occurs before flower initiation, either prior to or at inflorescence initiation. By the time the sepal primordia are initiated, flower sex type is easily distinguishable.

The B and C class organ identity genes are thought to be responsible for the sex determination in type II unisexual flowers. In spinach, two B class genes, SpAP3 and SpPI, are expressed in stamen development in male flowers, while they are not expressed in female flowers (Pfent et al. 2005). Recently, Hu et al. (2018) investigate the transcriptome assembly of reproductive organs of *P. volubilis*, and identified 195 and 2244 organ-specific genes in male and female flowers, respectively. They found genes specifically expressed in female flowers are enriched in plant hormone signal transduction pathways. However, studies of detailed expression patterns of these organ-specific genes in different developmental stages are necessary to identify responsible genes in relation to sex determination in *P. volubilis*.

### BA regulation of unisexual flower development

Sex expression in dioecious and monoecious plant species can often be altered by exogenous application of hormones such as auxins, gibberellic acid, and cytokinins (Korpelainen 1998). Sex modification has been reported in a number of monoecious and dioecious species in response to exogenous cytokinins treatments and the occurrence of hermaphroditic flowers and/or female flowers on the location of male flowers on the inflorescence and/or male individuals (Kahlem et al. 1975; Pan and Xu 2011; Fu et al. 2014; Pan et al. 2014, 2016). Kahlem et al. (1975) indicated that exogenous cytokinins induce the appearance of female flowers on the male individuals in *Mercurialis annua*. Cytokinin treatment promoting floral feminization in *Jatropha curcas*, as well as in *P. volubilis,* has also been reported (Pan and Xu 2011; Fu et al. 2014; Pan et al. 2014, 2016). Although those previous studies indicated exogenous applications of cytokinins could contribute to female flower formation, it is not clear when during development, and by what process, conversion of sex organ occurs.

In our present BA application experiments, many female flowers and intermediates of hermaphroditic flowers were induced in each BA-treated inflorescence. According to our findings, the extreme divergence in apical meristem between the male and female flowers occurs at the flower development stage 3 (female flowers, see Fig. 4a–c; male flowers, see Fig. 6b–d) and sex organs initiate at the flower development stage 4 (gynoecium initiation, see Fig. 4d; stamen initiation, see Fig. 6e) in a *P. volubilis* plant. When the exogenous BA began to take effect on the male flower meristem before or just at the early stage 4 of male flower development without any stamen primordia initiation, the whole male apical meristem could be converted to female meristem and initiate carpel primordia (see Fig. 8a, b), thus this flower meristem could develop into a normal female flower (see Fig. 8c–e). When the cytokinins began to take effect at the later stage 4 male flower development with more or less stamen primordia initiation (see Fig. 8f, k), the central apical meristem that undifferentiated into stamen primordia could initiate carpel primordia (see Fig. 8g, l), thus this flower meristem could develop into hermaphroditic flower (see Fig. 8h, j, m, o). The application of exogenous BA on the inflorescence at the early flower development stages shown in this study suggests that cytokinin is necessary for carpel induction and/or female flower development in *P. volubilis*. The function of cytokinins only performed on the undifferentiated apical meristem of male flowers. The cytokinins could modify the fate of the apical meristem of male flowers and promote the formation of carpel primordia. Moreover, these observations demonstrated that cytokinins seem to promote the pistil initiation rather than induce bisexual flower development in *P. volubilis*.

The effects of cytokinin on sexual modification in *P. volubilis* were similar to that in *Mercurialis annua*, which also has type II flowers. In dioecious *M. annua*, exogenous cytokinins induced female flowers on male plants and auxin induced male flowers on female plants (Hamdi et al. 1987; Louis et al. 1990). The floral meristem is thought to be sexual bipotent due to its sex expression can be altered by exogenous auxin and cytokinins (Louis 1989; Durand and Durand 1991). Talamali et al. (2003) also indicated the flower development of *Atriplex hamilus* (Chenopodiaceae) is remarkably plastic and endogenous, and environmental influences can determine the fate of the floral meristem in this species. Sex reversal by hormone application indicates genes required for the initiation and development of reproductive organs are functional but suppressed, possibly through a signal transduction mechanism that modifies endogenous levels of hormones (Dellaporta and Calderon-Urrea 1993). This supposition is in keeping with the recent work that indicated that genes regulating hormone metabolism play an essential role in sex determination in this species and *Jatropha curcas* (Chen et al. 2014; Pan et al. 2014; Fu et al. 2018). Additionally, very young inflorescences were used for cytokinin treatment experiments in this study, and the female flowers could be observed after 12 days suggesting that pre-evocation stages are likely to be critical. The longer treatment time of cytokinins in inflorescence could induce more female flowers. We hypothesized that the increased treatment time of exogenous cytokinin would result in an increase in cell division and carpel initiation and thus were more causal in the formation of female flowers in *P. volubilis*.

Dioecious plants, such as melons and kiwifruit, are currently under investigation to identify factors responsible for sex determination and flower organ identity in unisexual plants. Monoecious plants are seldom used in these studies. Monoecious plants like *P. volubilis* with distinguished unisexual flowers occur at different position of the same inflorescence and no rudimentary organs of opposite sex during unisexual flower development are potential model systems for such studies. According to our results, sexual determination can probably be assigned to a *P. volubilis* flower prior to or at the time of inflorescence initiation, and sexual differentiation certainly after sepal initiation. Use of *P. volubilis* in such studies would need to distinguish flower sex type as early as possible. Furthermore, we have proved cytokinin could take effect on feminization at the very earlier flower developmental stage in this species. To improve fruit yield of *P. volubilis* by increasing the number of female flowers, the plant growth regulator (BA) should be applied as early as possible, when inflorescence initiating.

#### Author contribution statement

ZFX designed the study and revised the manuscript. YL drafted the manuscript and prepared the figures. BZP performed BA treatment. CXY performed SEM. LL performed light microscope observation. All authors discussed the results and commented on the manuscript.
